# Comparison of conventional and digital workflow for dental rehabilitation with a novel patient-specific framework implant system: an experimental dataset evaluation

**DOI:** 10.1186/s40729-022-00405-7

**Published:** 2022-01-24

**Authors:** Simon Spalthoff, Mandy Borrmann, Philipp Jehn, Björn Rahlf, Nils-Claudius Gellrich, Philippe Korn

**Affiliations:** Department of Oral and Maxillofacial Surgery, Hannover Medical School, Carl-Neuberg Strasse 1, 30625 Hannover, Germany

**Keywords:** Temporary dental prosthesis, Implant, Tumor, Digitally constructed, Edentulous, Dental arches

## Abstract

**Purpose:**

This study aimed to evaluate the efficiency of a digital workflow by comparing the accuracy of prosthetic teeth positioning between virtual standard-size digitally constructed and conventional dental laboratory-fabricated prostheses.

**Methods:**

Twenty-five computed tomography datasets with a dentate upper jaw were selected after applying inclusion criteria to 100 random datasets obtained from the institutional library, and partially edentulous maxillae were constructed virtually. Digital datasets of temporary prostheses were fabricated on these virtually constructed edentulous maxillae in two ways: one dataset comprised prostheses that were fabricated conventionally using prosthetic teeth and wax in the dental laboratory and then scanned using a model scanner, whereas the other dataset was designed virtually using standardized virtual dental arches. The digital datasets of both prostheses were compared for differences at six dental-based measurement points with the original patient dentition.

**Results:**

Overall, the conventional design pathway was more accurate than the digital one (conventional 2.915 ± 1.388 mm, digital 3.609 ± 2.052 mm, *P* < 0.001). However, when all six measurement points were evaluated individually, only three points showed significant differences in the tooth positions. Compared with the original dentition, the deviations were less in the anterior teeth region than in the molar region, fulfilling the esthetic expectations of the patients. Standardized virtual dental arches were practically adequate because virtual reconstruction of every edentulous case using these virtual arches was possible without any additional modifications.

**Conclusion:**

It is possible to fabricate clinically acceptable temporary prostheses using a comprehensive digital workflow based on standardized digital dental arches.

## Background

**Fig. 1:**
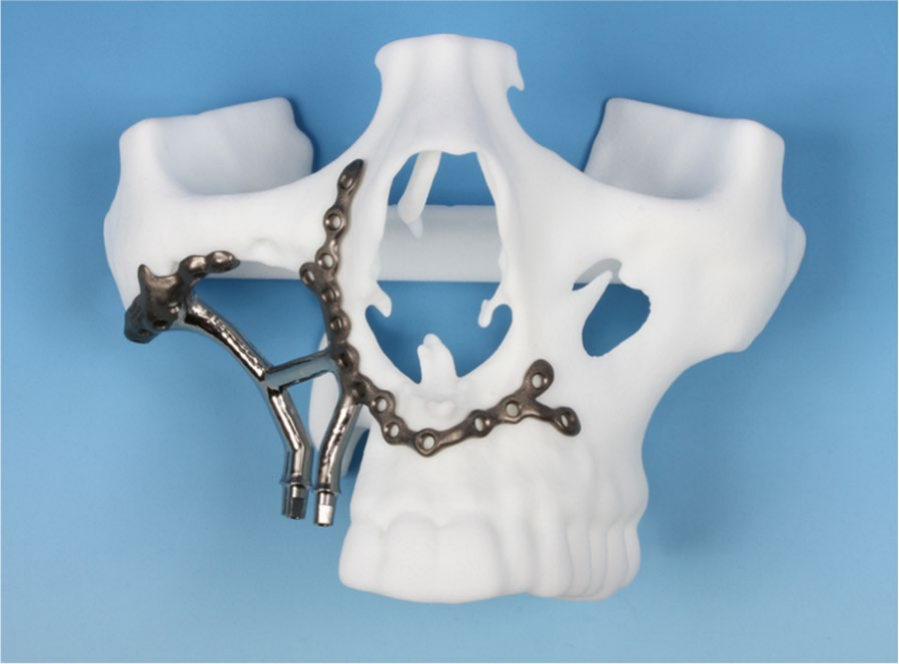
Patient-specific preprosthetic intended for the reconstruction of a major maxillary defect after tumor resection. Placed on the plastic model; Individual Patient Solution Implants^®^ Preprosthetic, KLS Martin Group, Tuttlingen, Germany

In 2017, Gellrich et al*.* proposed a novel framework implant system composed of prosthodontic-driven backward-planned implant posts and a wireframe-style framework customized for primary multivector fixation to the bony surface at the recipient site. The two components are digitally fused and manufactured as a single-piece implant by selective laser melting. To date, these patient-specific implants have been used in 60 patient cases by Gellrich et al*.* in Hannover (Fig. 1) [1, 2].

In 2020, Jehn et al. were the first to determine oral health-related quality of life (OHRQoL) after dental rehabilitation using this novel implant system. Their results showed improved OHRQoL in patients with severe bone deficiencies post-tumor resection, especially when the implants were combined with fixed dental prostheses [3]. In general, fixed prostheses offer better OHRQoL than other types of dental prostheses [4, 5].

The absence of teeth in general leads to problems related to esthetics, chewing, and speech, which results in a decrease in OHRQoL [6]. Therefore, immediate dental rehabilitation with a temporary prosthesis after implant placement is of utmost importance to maintain the well-being of patients [7]. Additionally, following oral cancer therapy, which often includes widespread tumor resections and microvascular soft-tissue reconstructions, temporary prostheses usually serve another purpose: they contour the soft tissues before rehabilitation with fixed prostheses; [8] help separate the anatomical units and prevent soft-tissue collapse.

To standardize the individual planning process for placement of the patient-specific framework implants developed by Gellrich et al*.* [1, 2], the authors of the present study developed a comprehensive digital workflow beginning from prosthodontic-driven backward planning to the computer-aided design (CAD)/computer-aided manufacturing (CAM) construction of temporary prosthesis with possible fabrication of permanent prosthesis using CAD/CAM.

To evaluate the efficiency of this digital approach, this study aimed to compare the accuracy of teeth positioning between the virtual standard-size digital dataset of temporary prostheses and conventional dental laboratory-fabricated digital datasets of temporary prostheses. Additionally, the conceptual viability of the standard-size virtual dental arches was evaluated.

## Methods

### Ethical approval

This experimental study was approved by the Ethics Committee of the Hannover Medical School (approval number 9815_BO_K_2021). The 1964 Helsinki Declaration and its later amendments or comparable ethical standards were complied within in this study. All patients provided written consent for the use of their data.

### Computed tomography (CT) data selection

We randomly selected 100 datasets from the radiographic records of the Department of Oral and Maxillofacial Surgery at the Hannover Medical School. The datasets were selected by chance out of an unsorted file folder with several thousand datasets with oral and maxillofacial disease patterns. These datasets were screened in alphabetical order until 25 datasets matching the following inclusion criteria were selected for the study:

- a CT scan with at least a 1-mm slice thickness
- complete maxillary dentition up to the first molar
- minimal artifacts due to metallic restorations or other structures
- written consent for the scientific usage of datasets by the respective patients.

All included datasets were anonymized and randomized using serial numbering.

### Digital workflow

**Fig. 2:**
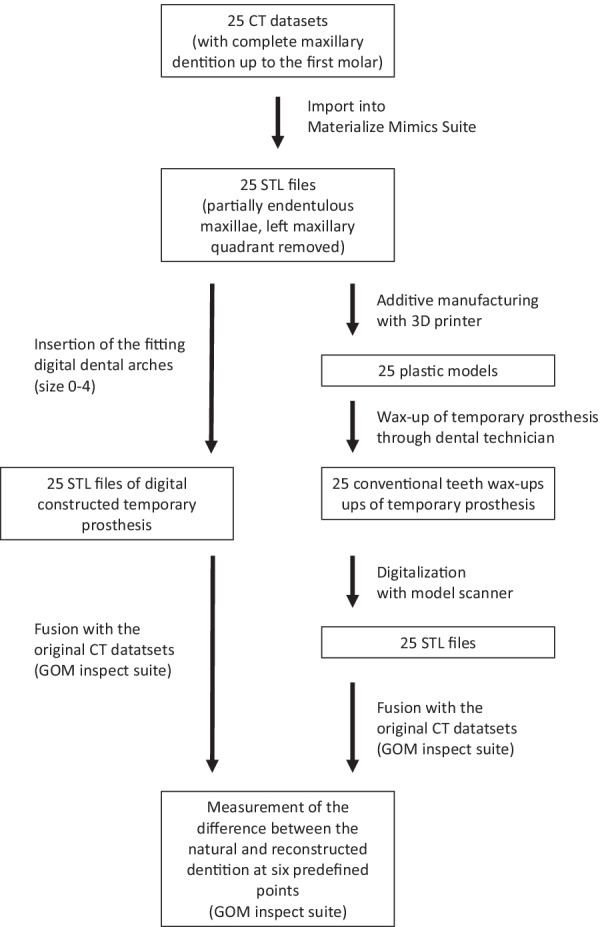
Flow chart of comprehensive digital workflow

**Fig. 3:**
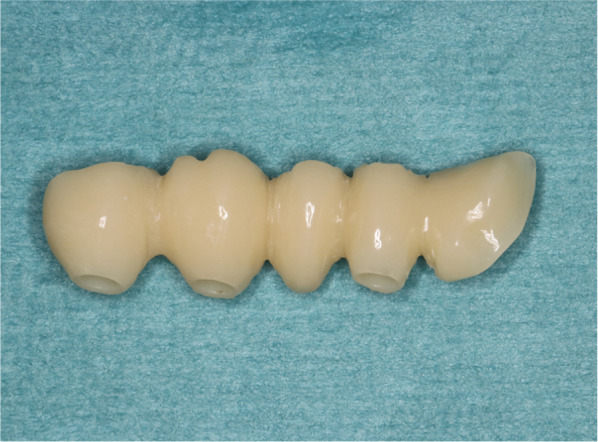
Digitally planned and additive manufactured temporary dental prosthesis. Resin was used for the individual patient-specific implant preprosthetic system shown in Fig. 1

A comprehensive digital workflow (Fig. 2) was prepared for the clinical application with the Individual Patient Solution Implants^®^ Preprosthetic (KLS Martin Group, Tuttlingen, Germany). Digital dental arches aligned to the sizes of commercially available impression trays (sizes 1–5 and XS-XL) were designed. The dental arch size of the patient reconstructed using this implant system was measured in advance, and a suitable temporary prosthesis was fabricated corresponding to the implant system. The material of the temporary prosthesis can be chosen according to the indication and expectation of the patient [e.g., titanium or plastics (Fig. 3)]. For this study, a simplified protocol was used, and only the digital datasets of the temporary prostheses were used for all analyses.

### Preparation of virtual models

**Fig. 4:**
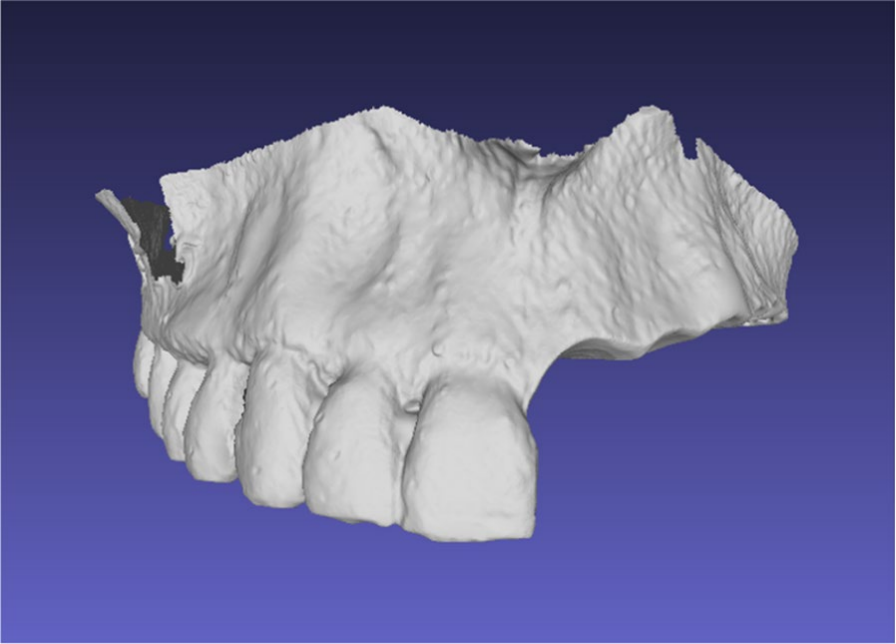
Virtual partially edentulous maxilla

All 25 datasets were imported using the DICOM format into the Materialize Mimics Suite software (Materialize, Leuven, Belgium). Afterimage smoothing and segmentation, individual arch data were separated, if necessary, and the mandibular arch data were discarded. The left maxillary quadrant dentition was virtually removed, and the resulting partially edentulous maxilla image was exported as a standard tessellation language (STL) file (Fig. 4).

### Fabrication of temporary prosthesis in dental laboratory

**Fig. 5:**
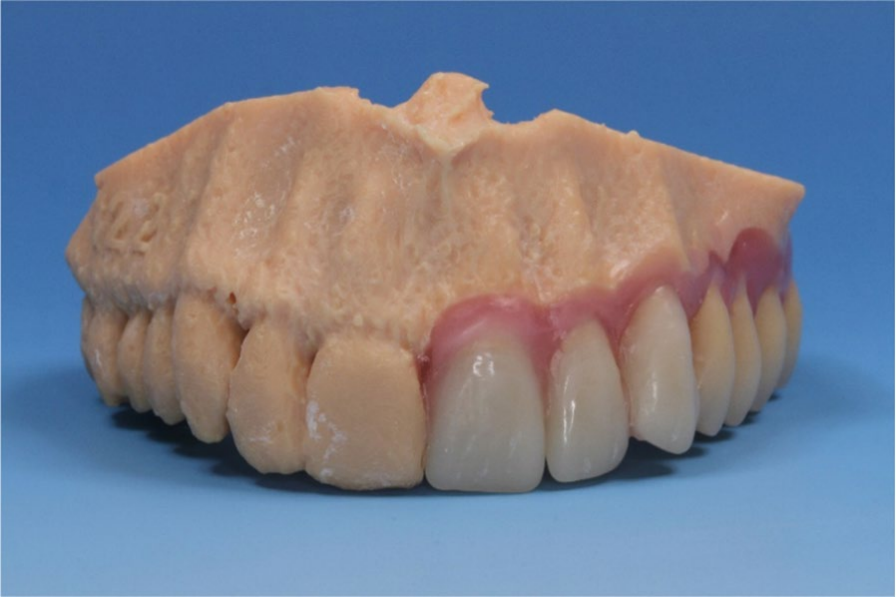
Conventional teeth wax-up of the temporary prosthesis

**Fig. 6:**
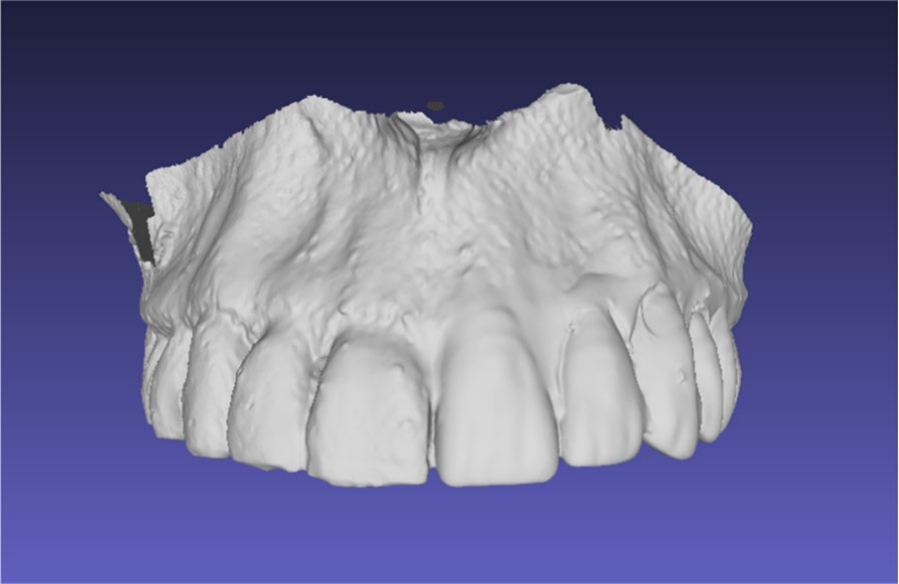
Digitalized conventional teeth wax-up of the temporary dental prosthesis

Three-dimensional models of the selected 25 partial edentulous maxillae were printed using additive manufacturing with a 3D printer (Ultimaker BV, Utrecht, Netherlands). Afterward, teeth wax-up of the temporary prosthesis was performed by a dental technician using acrylic resin teeth and wax (Fig. 5). The teeth were positioned according to the tooth-bearing quadrant of the upper jaw. No occlusion contacts were planned. Later, these 3D models with waxed-up teeth were digitalized using a conventional model scanner (S600 Arti, Zirkonzahn GmbH, Gais, Switzerland) (Fig. 6).

### Manufacturing of the digital temporary prosthesis

**Fig. 7:**
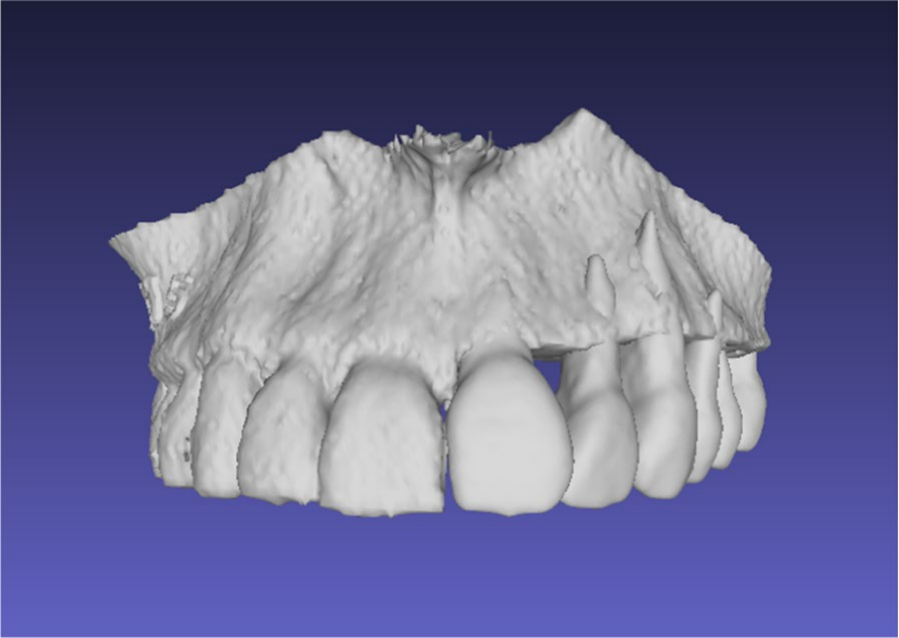
Digitally constructed temporary prosthesis

All 25 partially edentulous maxillary STL files were imported into the Geomagic Freeform^®^ software (3D Systems, Rock Hill, South Carolina, USA). The image surfaces were smoothed, and virtual dental arches of the fitting sizes were inserted in the edentulous space. These virtual dental arches were replicated from one healthy artifact-free dental CT dataset several years ago and virtually adapted to different standard impression tray sizes (0–4), with 0 being the smallest and 4 being the largest size of dental arches. These virtual dental arch sizes were developed to fit most patients’ original dental arch sizes. The fitting size digital dental arches were chosen, and the dental arches were cut to the correct length to fit the defects. Then the cut dental archer was inserted digitally to reconstruct the patients’ dental arches. The resulting images of reconstructed maxillae were exported as STL files (Fig. 7).

### Comparison of conventionally fabricated and digitally constructed temporary prostheses with the original dentition

**Fig. 8:**
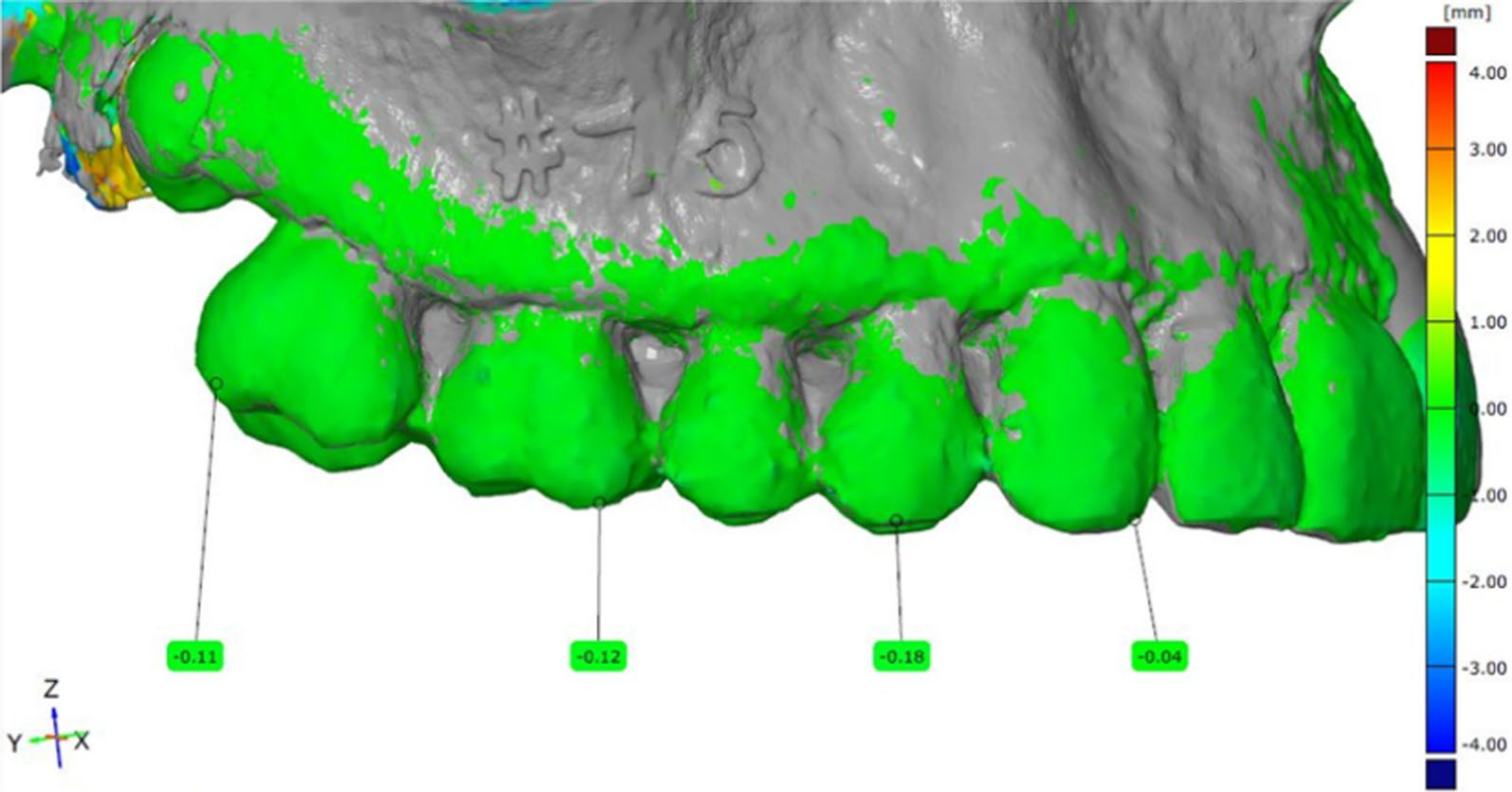
Heat map of the superimposed standard tessellation dental prosthesis and original dentition language files

The respective STL files of conventionally and digitally constructed dentitions were superimposed over the original STL files of the unmodified maxillae (original teeth still in place). This superimposition was performed using the unmodified right maxillary quadrant as a guide. A heat map was used to monitor the superimposition process (Fig. 8).

After sufficient fusion, the corresponding predefined six points (incisal edges of 22 and 23, buccal and palatal tooth cusp tips of 24, and mesiobuccal and distopalatal tooth cusps of 26) were marked on the natural and reconstructed dentition. The difference between the original and reconstructed dentition was measured at three different points (*z, x, y*), defining vector *L*. The length of the connecting vector *L* was calculated in millimeters using the following formula:

$$\left( {\left| {\vec{L}} \right| = L = \sqrt {L_{x}^{2} + L_{y}^{2} + L_{z}^{2} } } \right)$$

**Fig. 9:**
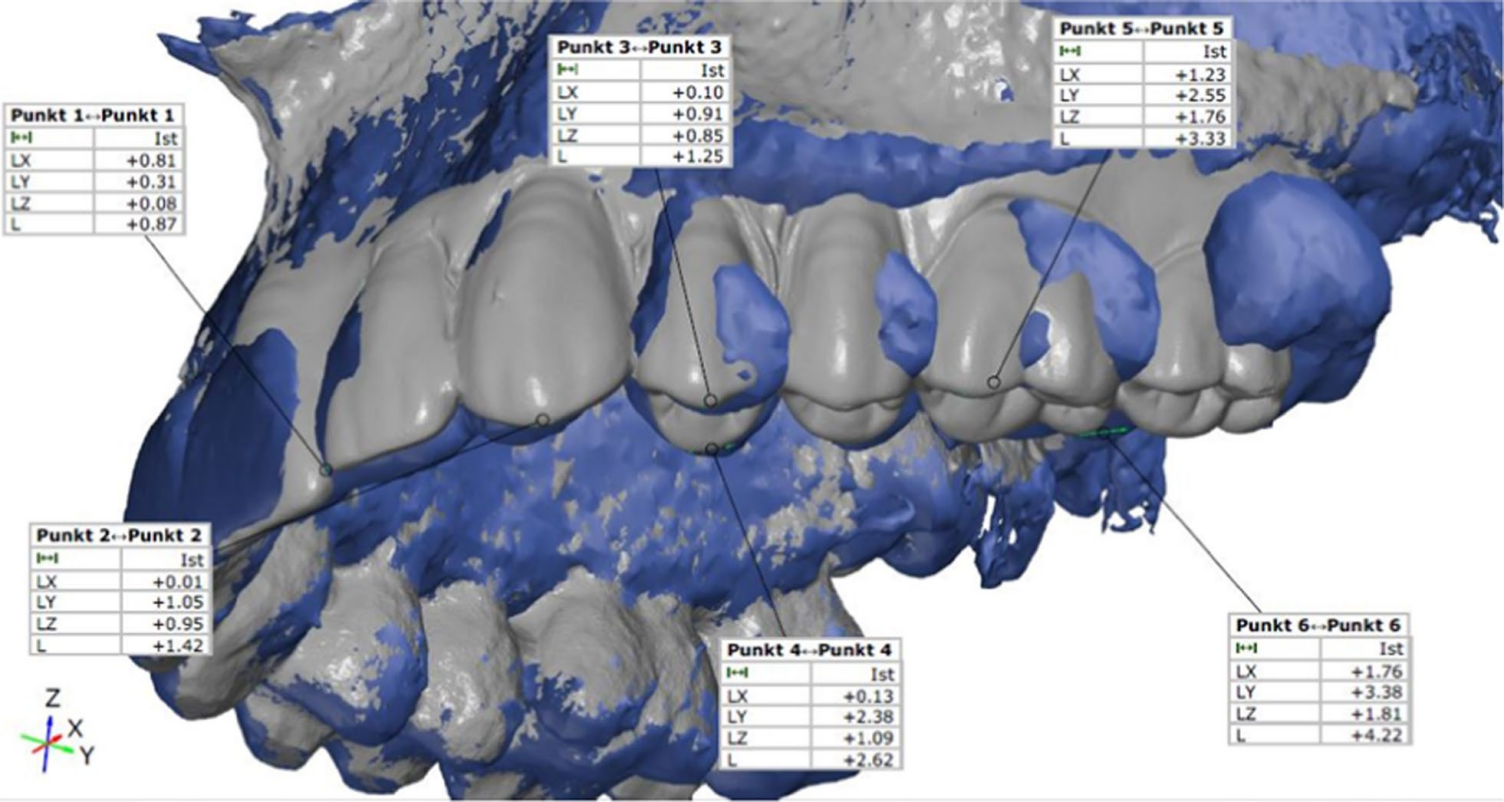
Difference (vector L) between the teeth positions in the original dentition and conventional/digital dental prosthesis measured in millimeters at six different predefined dental points

The resulting *L* values were used for further analysis (Fig. 9). For all 3D analyses, GOM inspect suite (GOM inspect 2019, GOM GmbH, Braunschweig, Germany) was used.

### Statistical analysis

The obtained data were analyzed using SigmaPlot 13.0 software (Systat Software Inc., San Jose, California, USA). The Shapiro–Wilk-Test was used to test for normality. The paired *t*-test was performed for normally distributed values, the Wilcoxon signed-rank test for non-normally distributed values to check for significance, and a *P*-value of < 0.05 was considered statistically significant.

## Results

The accuracy of the digital workflow was sufficient for obtaining the necessary measurements. The standard-size virtual dental arches accurately fitted the virtually created maxillary edentulous spaces without any need for additional modifications. Virtual dental arches of size 3 (*n* = 6) and size 4 (*n* = 19) were used for fabricating the datasets of the digitally constructed temporary prostheses.

**Fig. 10:**
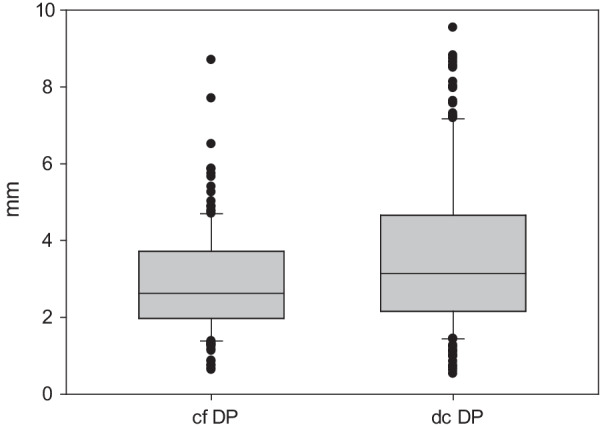
Box-plot of the overall differences. Differences between the conventionally fabricated dental prosthesis and the original dentition (cf DP) versus the difference between the digitally constructed dental prosthesis and the original dentition (df DP) in millimeters

Comparing the overall results of all measurements between the conventionally and digitally constructed temporary prostheses, the conventional prosthesis was more accurate in reproducing the original teeth position of the patients (overall difference to the original dentition: conventional 2.915 ± 1.388 mm, digital 3.609 ± 2.052 mm, *P* < 0.001) (Fig. 10).

However, analysis of the individual L values at six different measurement points showed varying results. For three out of six measurement points ( difference to the original dentition point of measurement *22*: conventional 1.691 ± 0.513 mm, digital 1.918 ± 0.655 mm, *P* = 0.146; *23*: conventional 2.528 ± 0.171 mm, digital 3.397 ± 0.304 mm, *P* = 0.031; *24 buccal*: conventional 2.814 ± 0.842 mm, digital 3.997 ± 1.891 mm, *P* = 0.581; *24 palatal*: conventional 3.744 ± 1.169 mm, digital 3.837 ± 1.468 mm, *P* = 0.026; *26 mesiobuccal*: conventional 3.116 ± 1.863 mm, digital 4.183 ± 2.597 mm, *P* = 0.013; *26 distopalatal*: conventional 3.600 ± 1.562 mm, digital 4.320 ± 2.611 mm, *P* = 0.226) there was no significant difference between the vector lengths of the conventionally and digitally constructed temporary prostheses (Table 1).

**Table 1:** Comparison of differences in teeth positioning between conventionally fabricated and digitally constructed dental prostheses with the original dentition

Point of measurement	Type of prosthesis	N	Mean* Median^+^ (mm)	Standard deviation (mm)	25%	75%	*P*-value
22	Conventional	25	1.691^*^	0.513			0.146*
	Digital	25	1.918^*^	0.655			
23	Conventional	25	2.528^*^	0.857			0.031*
	Digital	25	3.397^*^	1.522			
24 buccal	Conventional	25	2.820^+^		2.210	3.485	0.026^+^
	Digital	25	3.330^+^		2.725	4.895	
24 palatal	Conventional	25	3.744^*^	1.169			0.581*
	Digital	25	3.997^*^	1.891			
26 mesiobuccal	Conventional	25	2.520^+^		2.030	3.795	0.013^+^
	Digital	25	3.910^+^		2.070	5.825	
26 distopalatal	Conventional	25	3.600^*^	1.562			0.226*
	Digital	25	4.320^*^	2.611			
Total	Conventional	150	2.625^+^		1.967	3.720	< 0.001^+^
	Digital	150	3.140^+^		2.158	4.662	

*Paired *t* test; ^+^ Wilcoxon signed-rank test

The reconstruction in the molar teeth region was less precise than in the anterior teeth region (2.8–4.3 versus 1.7–3.4 mm)

## Discussion

Several digital workflow systems have been described in the literature for fabricating temporary (immediate) and permanent prostheses using intraoral scanners, cast model scanners, and CAD/CAM software [9–11]. However, a simplified method was used in the present study with the aim of placing the temporary prosthesis out of occlusal contact.

The inaccuracy in the positions of the prosthetic teeth in comparison with those in the original dentition ranged from 1.7 to 3.7 mm and 1.9 to 4.3 mm in the conventionally and digitally constructed temporary prostheses, respectively. The overall positioning was significantly better in the conventionally constructed prosthesis than in the digital one (*P* < 0.001). This might be due to the fact that the individual tooth position is better adaptable manually by the dental technician than by using only 5 standard size digital dental arches. Nevertheless, when the measurement points were evaluated individually, three out of six points showed no significant differences in the results between the conventional and digital approaches. Both methods are rather inaccurate, as shown in Fig. 1 (outliers in the box plot). In some cases, the position of the original dentition is almost reached, and in other cases, it is missed by several millimeters. This could be due to some cases with abnormalities in the original dental arches, which we did not consider. In real patient cases, the temporary prosthesis would ideally be orientated at the dentition prior to teeth removal, for example, using older plaster models.

Nevertheless, in our opinion, both methods can be used to construct temporary prostheses when using the described patient-specific framework implant system. Considering that the tooth positions were more accurate in the anterior teeth region (1.7–3.4 mm) than in the molar teeth region (2.8–4.3 mm), it can be concluded that the esthetically important anterior teeth region was more adequately reconstructed in accordance with the patient’s expectations. The inaccurate reconstruction of the original dentition in the molar region using both methods is clinically less significant as temporary prostheses have minimal esthetic function in that region and are generally intended for the contouring of the surrounding soft tissues. The technique of forming soft tissues using temporary prostheses has been described in conventional implant prosthetics [7, 8]. The soft tissues often pose a major challenge for implant-supported prosthetic rehabilitation following the microvascular reconstruction of tumor-induced jaw defects [12]. Post-tumor resection cases rehabilitated using conventional dental implants often show peri-implantitis [13, 14]. A major advantage of this new system is the relative resilience against peri-implantitis since the implant fixation to the bone is usually far away from the implant posts. So even if peri-implantitis cannot be controlled by cleaning the implant posts and optimal oral hygiene, it does not lead to loosening of the implants [1, 2].

Furthermore, occlusal contact was not planned in either of the temporary prostheses (conventional or digital); hence, static and functional occlusion analyses were not considered in this study. However, the digital process used here for the fabrication of temporary prosthesis can be further developed to fabricate permanent prosthesis. One possible optimization might be the use of different digital dental arches not only in size but also in the forms of teeth to better resemble the original dentition. Concepts of complete digitally planned dental restorations have already been proven by several authors [15, 16]. Charette et al*.* combined a complete fixed implant-supported dental prosthesis of the mandible with a conventional complete removable dental prosthesis of the maxilla. They used CAD/CAM with adjusted occlusion in the centric condylar position, which provided satisfactory results for the patient [17]. Commercially available CAD/CAM software solutions are available in the market for digital designing and fabrication of complete dentures [9]. The combination of different imaging techniques, such as CT and 3D-surface information acquired by a face scanner, could help in designing dentures using CAD/CAM software solutions. These dentures will be not only technically efficient but also esthetically acceptable [18].

Standard sizes 3 and 4 of the digital dental arches were used in the present study, with size 4 (*n* = 19) being used more often. Therefore, it could be possible to reduce the number of digital dental arches further. Nevertheless, it was possible to reconstruct every case with the available virtual dental arches, demonstrating that the concept of impression tray-oriented sizes is practically adequate. The digital workflow described in this manuscript was specially designed for this patient-specific framework implant mentioned in the Digital Workflow paragraph. It has been used until now mainly to contour the soft tissues and for esthetic reasons. In the future, it might be possible to implement a digital workflow for the patient-specific framework to create permanent prosthesis with an adapted occlusion using the digital techniques by other groups as discussed above. Until then, our standard digital arches provide an easy and quick way to fabricate temporary dental prosthesis, which proved to be sufficient for temporary reconstruction in cases with patient-specific framework implants.

## Conclusions

It is possible to fabricate clinically acceptable temporary prostheses using a comprehensive digital workflow based on standardized digital dental arches. Moreover, the esthetic outcomes of the digitally planned prosthesis in the anterior teeth region were comparable to those of the conventional prosthesis. In the future, this digital workflow may be optimized to fabricate permanent prostheses using a single-step procedure in combination with a patient-specific framework implant system.

## Data Availability

Not applicable.

## Abbreviations

OHRQoL: Oral health-related quality of life
CAD: Computer-aided design
CAM: Computer-aided manufacturing
CT: Computed tomography
STL: Standard tessellation language
